# Supplementary material for: Arterial Blood Gas Analysis for Survival Prediction in Pediatric Patients with Out-of-Hospital Cardiac Arrest
Source: J Pers Med. 2023 Jun 28;13(7):1061. doi: 10.3390/jpm13071061 (PMC10381305; doi:10.3390/jpm13071061)

Supplementary Figure S1. Cut-off values of arterial blood gas analysis variables for survival to hospital admission using receiver operating characteristic curves. AUC, area under the curve. (A) pH, (B) PaCO2, (C) PaO2, (D) Lactate.

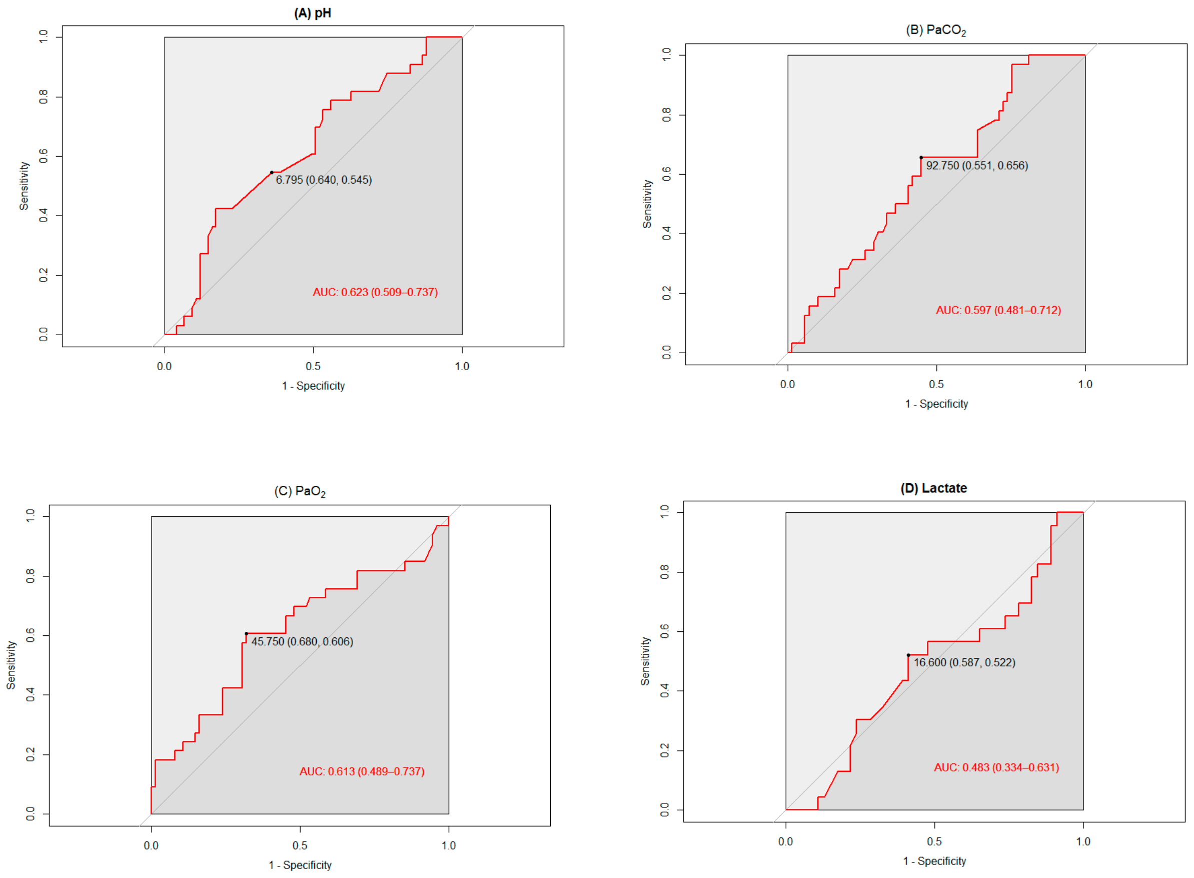

Supplementary Figure S2. Cut-off values of arterial blood gas analysis variables for survival to discharge using receiver operating characteristic curves. AUC, area under the curve. (A) pH, (B) PaCO2, (C) PaO2, (D) Lactate.

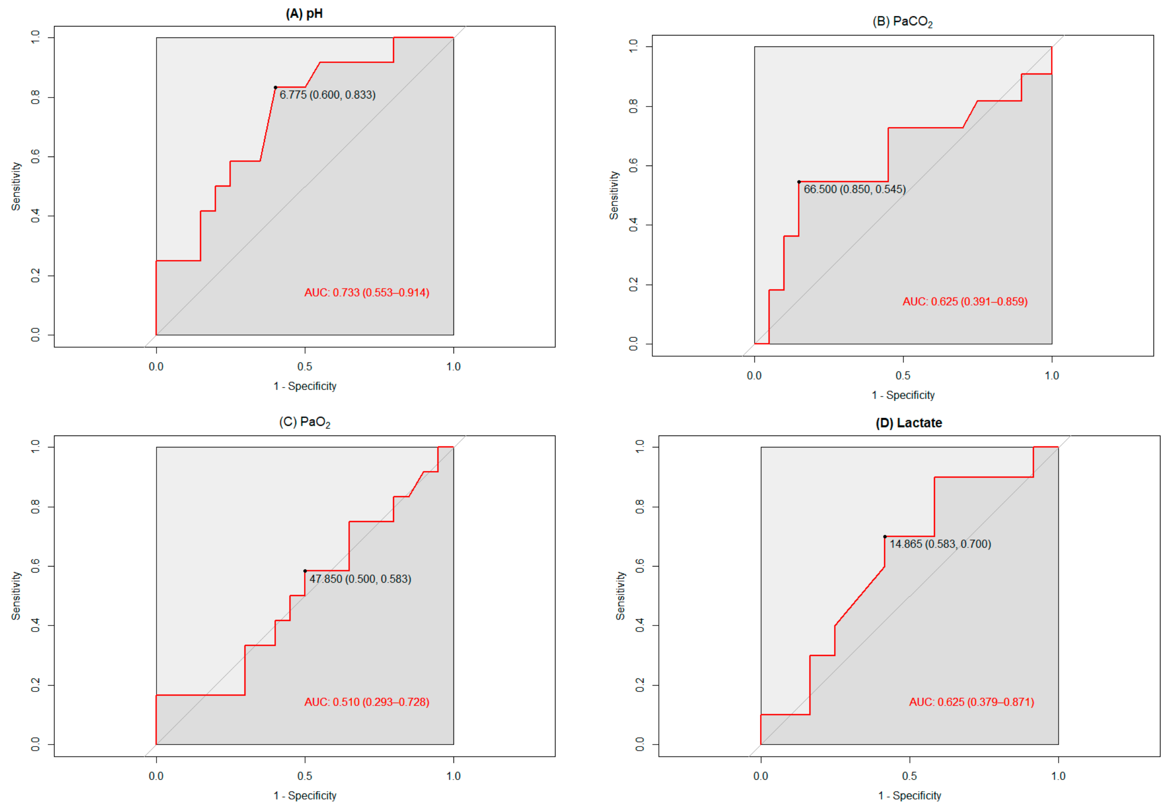

Supplement: Supplementary file 1 [file jpm-13-01061-s001.zip › JPM_Supplement figures_ABGA OHCA.pdf]
